# Kidney Drug Transporters in Pharmacotherapy

**DOI:** 10.3390/ijms24032856

**Published:** 2023-02-02

**Authors:** Joanna Łapczuk-Romańska, Maria Droździk, Stefan Oswald, Marek Droździk

**Affiliations:** 1 Department of Pharmacology, Pomeranian Medical University, Powstancow Wlkp 72, 70-111 Szczecin, Poland; 2 Medical Faculty, Medical University of Lodz, Tadeusza Kościuszki 4, 90-419 Lodz, Poland; 3 Institute of Pharmacology and Toxicology, Rostock University Medical Center, 18051 Rostock, Germany

**Keywords:** drug transporters, kidney

## Abstract

The kidney functions not only as a metabolite elimination organ but also plays an important role in pharmacotherapy. The kidney tubule epithelia cells express membrane carriers and transporters, which play an important role in drug elimination, and can determine drug nephrotoxicity and drug–drug interactions, as well as constituting direct drug targets. The above aspects of kidney transport proteins are discussed in the review.

## 1. Introduction

The kidney is a key organ engaged in the elimination of hydrophilic molecules of endogenous and exogenous origin. Renal elimination of drugs involves complementary processes, to which glomerular filtration and tubular secretion and reabsorption can contribute [1]. Excreted compounds reach the nephron lumen via glomerular filtration or passive, facilitated or active transmembrane transport through tubule epithelial cells. The tubule cells are endowed with carriers (facilitating substrate drug transport) and transporters (which are energy dependent), which are expressed on basolateral or apical membranes along the nephron, i.e., in proximal, distal and collecting tubule epithelia.

**Figure 1:**
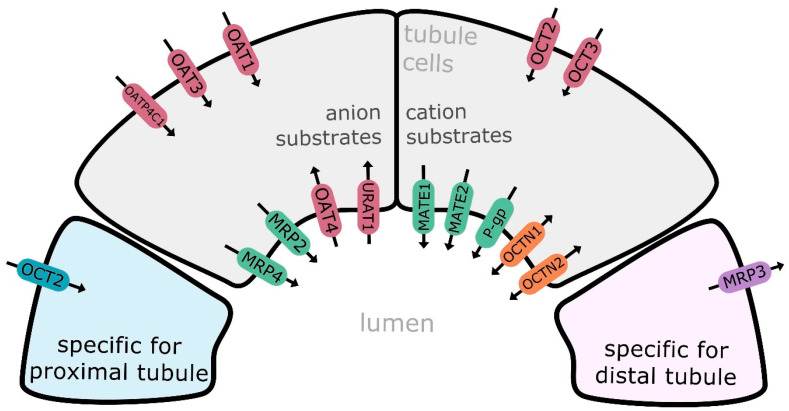
Major drug transporters in the kidney renal epithelial cell: MATE1—multidrug and toxin extrusion protein 1; MATE2/*SLC47A2*—multidrug and toxin extrusion protein 2; MATE2-K—multidrug and toxin extrusion protein 2 kidney-specific; MRP2—multidrug resistance-associated protein 2; MRP3—multidrug resistance-associated protein 3; MRP4—multidrug resistance-associated protein 4; P-gp—multidrug resistance protein 1/P-glycoprotein; OAT1—organic anion transporter 1; OAT3—organic anion transporter 3; OAT4—organic anion transporter 4; OATP4C1—organic anion transporting polypeptide 4C1; OCT2—organic cation transporter 2; OCT3—organic cation transporter 3; OCTN1—organic cation/carnitine transporter 1; OCTN2—organic cation/carnitine transporter 2; URAT1—urate transporter 1. Efflux transporters/carriers highlighted in green/violet, influx carriers in red/bleu and bidirectional carriers in orange.

Transport proteins are divided into two major superfamilies, i.e., ATP-binding cassette transporters (ABC, comprising about 50 members, classified into seven families) and solute carriers (SLC, with more than 400 membrane proteins allocated into over 60 families) [2,3]. Transporters of the ABC superfamily are mainly engaged in efflux activities, and the list of major representatives includes multidrug resistance protein 1/P-glycoprotein (MDR1, P-gp, *ABCB1*), and multidrug resistance-associated protein 2 (MRP2, *ABCC2*), 3 (MRP3, *ABCC3*), and 4 (MRP4, *ABCC4*). SLC carriers mediate cellular influx and/or efflux of substrate molecules, operating in a gradient-dependent manner. Organic anion transporting polypeptide 4C1 (OATP4C1, *SLCO4C1*), organic anion transporter 1 (OAT1, *SLC22A6*), 2 (OAT2 *SLC22A7*), 3 (OAT3, *SLC22A8*), and 4 (OAT4, *SLC22A11*), organic cation transporter 2 (OAT2, *SLC22A2*), organic cation/carnitine transporter family members OCTN1 (*SLC22A4*) and OCTN2 (*SLC22A5*), multidrug and toxin extrusion proteins (MATE1/*SLC47A1*; MATE2/*SLC47A2;* MATE2-K/*SLC47A2*), peptide transporters 1 and 2 (PEPT1/*SLC15A1*; PEPT2/*SLC15A2*), equilibrative nucleoside transporters 1 (ENT1/*SLC29A1*) and 2 (ENT2/*SLC29A2*), urate transporter 1 (URAT1, *SLC22A12*), and sodium-glucose co-transporter 2 (SGLT2/*SLC5A2*) belong to the major representatives of SLC carriers in the kidney. The distribution of transporters along nephron seems to be partially segment specific (likewise in other segmental organs, e.g., gastrointestinal tract), and proximal, distal and collecting duct epithelium can possess different panels of membrane carriers and transporters, e.g., OCT2, GLUT9 and SGLT2 are examples of carriers exclusively expressed in the kidney proximal tubule cells, whereas MRP3 expression was defined in distal tubule cells [4,5] (Figure 1). However, it should be stated that the best characterized are transporters located in the kidney proximal tubule [5,6,7]. 

In the kidney, SLC carriers (OCTs, OATs, MATEs) are more abundantly expressed than the ABC transporter family members (P-gp, MRPs, BCRP). OAT1, OAT3, OCT2 and MATE1 belong to the most expressed SLC carriers, and P-gp, MRP2 and MRP4 represent the most abundant ABC transporters [8,9]. The drug carriers and transporters function in a coordinated manner, which allows transmembrane shift of cation, anion or zwitterion compounds. SLC carriers, which include anions (OATs), cations and zwitterions (OCTs, OCTNs, MATEs), function in a facilitative manner, shifting substrates along electrochemical gradients, and can provide both uptake and efflux activity. In their less frequent active mode, SLC transporters provide transport activity against a gradient of a substrate by coupling it to the electrochemical gradient of a co-transported ion (e.g., Na^+^, H^+^) or solute. The ABC transporters, involved in renal handling of anions (MRPs) or cations (P-gp) operate primarily as active transporters that can transport substrates against their electrochemical gradients, utilizing energy generated from ATP hydrolysis [5].

In the kidney, several membrane transporters and carriers are of clinical importance. They constitute direct therapeutic targets, are involved in drug elimination, can function as a site for clinically significant drug–drug interactions (DDIs), and can be implicated in nephrotoxicity of therapeutic agents, as has been highlighted in statements by the Food and Drug Administration (FDA) and in the recommendations of the International Transporter Consortium [10,11]. The list of these transporters includes P-gp, OAT1, OAT3, OCT2, MATE1 and MATE2-K. It can also be stated that these carriers and transporters participate in the handling of endogenous substrates, with creatinine and uremic solutes/toxins being examples [12].

The major drug substrates of principal renal transporters and carriers are presented in Table 1.

In humans, the expression and function of renal drug transporters have mostly been characterized in a healthy state, but it is obvious that, like in other organs (e.g., liver, intestine), kidney pathological states involve dysregulation of membrane transporters. Kidney biopsy specimens from patients diagnosed with lupus nephritis, IgA nephropathy, focal glomerular sclerosis, membranoproliferative glomerulonephritis, membranous glomerulonephropathy, and mesangial proliferative glomerulonephropathy displayed a significant reduction in *SLC22A6* (OAT1) mRNA expression (and stable levels of *SLC22A8*, *SLC22A7* and *SLC22A11* expression). The downregulation of OAT1 showed significant correlation with cefazolin elimination rate constant [17]. Another clinical finding in support of this is the dysfunction of OATs in patients with severe renal dysfunction (creatinine clearance (CrCl) < 30 mL/min), who demonstrated decreased renal clearance of sulfate conjugate of morinidazole (substrate for OAT1 and OAT3) and glucuronide conjugates (substrates for OAT3) [18]. Critical analysis of clinical data for 33 OAT1 and OAT3 probes revealed that the decrease in OAT1/3 activity was not proportional to the changes in glomerular filtration rate (GFR), and was insufficient to represent the effects of severe chronic kidney disease (CKD) on unbound tubular secretion clearance. OAT1/3-mediated tubular secretory clearance was estimated to decrease by an additional 50% relative to the decline in GFR in severe CKD, whereas the change in active secretion in mild and moderate CKD was proportional to GFR [19]. In CKD, accumulation of uremic solutes (e.g., indoxyl sulfate, kynurenate, anthranilate) may also affect renal drug elimination via the transporter system. The interaction of uremic retention solutes with BCRP (hippuric acid, indoxyl sulfate, kynurenic acid) and MRP4 (indole-3-acetic acid, phenylacetic acid) was demonstrated. The inhibition of transport by uremic toxins occurred at clinically relevant concentrations, suggesting that this mechanism may contribute to an altered functional role of the transporters in CKD [20]. Many of the uremic toxins/solutes are also substrates of OATs, and those agents may potentially compete with drugs at transporter sites affecting residual tubular secretion in the failing kidney [21]. 

The molecular mechanisms regulating expression of renal transporters in kidney disease states are not entirely defined. The effect of uremic serum on drug transporter expression was defined in in vitro models. Exposure of human kidney-2 cells (HK-2, a proximal tubule cell model) to sera obtained from rats with CKD resulted in significant downregulation of the protein expression levels of OAT3, OATP1 and P-gp, whereas levels of MRP2, MRP4 and OATP2 were significantly upregulated [22]. These findings are in line with studies in animals with experimentally induced CKD. The kidney dysfunction was characterized by downregulation of uptake carriers (Oats) and increased function of efflux transporters (Mrp2, Mrp4, P-gp) in the organ [22,23,24,25].

The molecular mechanisms involved in the regulation of renal transporter expression in CKD are poorly understood. In the kidney, similar to in other organs, drug transporters can be regulated at transcriptional and post-transcriptional levels [26]. The available information shows that hepatic nuclear factor (HNF)1β and DNA methylation are engaged in the regulation of *SLC22A6* (OAT1) and *SLC22A8* (OAT3) expression [27,28]. *SLC22A2* (OCT2) in kidney cells (HEK293, renal cell carcinoma) can be regulated by methylation and acetylation of HNF4 and its promoter [29,30]. Another transcription factor, nuclear factor E2-related factor 2 (Nrf2), has been shown to regulate expression of *ABCB1* (P-gp) and *ABCG2* (BCRP) in human tubular epithelial cells [31]. Post-translational mechanisms are postulated to regulate OAT1 and OAT3 (phosphorylation, glycosylation, ubiquitination), OCT2 (phosphorylation, glycosylation) and P-gp (phosphorylation) [26]. However, the manner in which those regulatory mechanisms function in CKD has not been defined. Some CKD-related solute toxins or inflammatory factors have been demonstrated to regulate renal transporters. Parathyroid hormone (PTH) (a uremic toxin) reduced Mrp2 protein levels and activity through PKC signaling in killifish renal proximal tubules [32]. Tumor necrosis factor (TNF)-α, a potent mediator of inflammation involved in the pathophysiology of CKD, has been reported to increase P-gp gene and protein expression levels and efflux activity in renal proximal tubule epithelial cells. The P-gp upregulation seemed to involve toll-like receptor (TLR)4 activation and nuclear factor κB (NF-κB) translocation [33]. Another study revealed the involvement of Nrf2 in regulating Mrp2 and Mrp4, but in cisplatin-induced nephrotoxicity. This pathway is activated under oxidative stress conditions, and also operates in CKD [34]. However, these findings were derived from experimental studies, and information about the regulation of drug transporters in the human kidney still needs to be defined.

## 2. Kidney Transporters in Pharmacotherapy

Drug transporters and carriers in the kidney are intentionally, directly targeted to produce therapeutic effects. Drugs affecting sodium-glucose cotransporter 2 (SGLT2) and URAT1 carriers/transporters are major drugs used in clinical medicine modulating renal transport systems.

SGLT2 (*SLC5A2*) is not a typical drug carrier, but is a drug target. It is primarily expressed in segments 1 and 2 of the renal proximal tubule, and is responsible for the reabsorption of ≈ 90% of the filtered glucose load. Inhibition of glucose reabsorption entails other clinically important effects, i.e., reduction of albuminuria and GFR decline, decrease in glucose-coupled sodium reabsorption, normalization of solute delivery to macula densa, restoration of tubuloglomerular feedback, mitigation of afferent arteriole vasodilation [35,36]. Modulation of SGLT2 transporter function affects not only glucose handling and reduces glucose levels in blood, but also produces indirect effects on glomerular and tubular functions, and thus promotes natriuresis and reduced albumin excretion. These effects contribute to a reduction in blood pressure and fluid accumulation in the body. Drugs inhibiting SGLT2 were initially registered in the treatment of diabetes mellitus type 2 (canagliflozin, dapagliflozin, empagliflozin ertugliflozin), and afterwards, dapagliflozin was also used in treatment of diabetes mellitus type 1. However, the authorization holder for dapagliflozin withdrew the indication for type 1 diabetes mellitus in 2021 (EU, UK, USA). Subsequently, the drugs were demonstrated to be effective in the treatment of heart failure with reduced ejection fraction (HFrEF), leading to reduction in the combined endpoint of myocardial infarction, stroke, cardiovascular death, hospitalization from heart failure, and occurrence of renal failure in patients with known cardiovascular disease or at high risk of developing cardiovascular disease [37]. Dapagliflozin and empagliflozin are even indicated for the treatment of patients with heart failure without diabetes. More recently, it has been found that canagliflozin improves symptoms in patients with heart failure with preserved ejection fraction (HFpEF) [38], which seems to be a unique feature of this SGLT2 inhibitor, as all other medications have been shown to improve prognosis in those patients. SGLT2 inhibitors have also been shown to decrease the risk of renal disease progression, with a similar benefit in patients with and without atherosclerotic cardiovascular disease. The magnitude of benefit produced by SGLT2 inhibitors varied with baseline renal function, with greater reductions in hospitalizations for heart failure and smaller reductions in progression of renal disease in patients with more severe kidney disease at baseline [39]. The class of SGLT inhibitors also includes sotagliflozin, which combines inhibitory activity against SGLT2 and SGLT1, and is referred to as a dual SGLT inhibitor. SGLT1 is predominantly expressed in the apical membrane of enterocytes in the small intestine, where it is involved in absorption of glucose, but it also functions in the kidney. SGLT1 controls reabsorption of nearly 10% of the filtered glucose load in the renal proximal tubule segment [40]. Recently, the drug received approval in the treatment of heart failure in adult patients with type 2 diabetes [41]. Its application for the treatment of diabetes type 1 and 2 was suspended.

Uric acid is freely filtered in the glomerulus, and then undergoes reabsorption mediated by apical transporters engaged in urate handling located in the proximal tubules, along with a small fraction of it being transported into the filtrate in the distal segment of the proximal tubule. The amount of uric acid excreted is determined by activities of urate carriers and transporters, which act as both reabsorption and excretion transporters. The uptake function (from blood into tubule cells) is mainly provided by OAT1 and OAT3. The excretion of uric acid (from tubule cells to nephron lumen) is determined by URAT1 (major uric acid reabsorption transporter), OAT4 (reabsorption carrier), and MRP4, sodium-dependent phosphate transport protein (NPT1/*SLC17A1*) and BCRP (secreting uric acid from the cells to the lumen) [42].

The uric acid carrier, i.e., URAT1 (*SLC22A12*), is another example of the transporters being targeted by drugs for therapeutic effects. The URAT1 carrier is expressed on the luminal side of the proximal renal tubule, and is engaged in the absorption of uric acid from the renal tubule lumen to the epithelial cells [42]. In the kidneys, about 90% of the urate filtered in the glomeruli is further reabsorbed, and only 10% is excreted by the kidneys. URAT1 is the major regulator of uric acid reabsorption processes, and its dysfunction is associated with hyperuricemia. To date, other two transporters have also been found to be engaged in uric acid reabsorption, i.e., organic anion transporter 4 (OAT4, *SLC22A11*) and glucose transporter 9 (GLUT9, *SLC2A9*) [43]. URAT1 and GLUT9 are targeted by drugs, and the inhibition in patients with hyperuricemia is of therapeutic value. URAT1 function is modulated by lesinurad (USA, Europe) and dotinurad (Japan), which are selective urate reabsorption inhibitors (SURIs). Lesinurad inhibits URAT1 and organic anion transporter OAT4, and does not interact with GLUT9 nor OAT1 or OAT3 [44]. Reduced reabsorption rate of uric acid in the apical membrane entails downregulation of its systemic concentrations. Mean serum uric acid decreases at lesinurad dose of 200 mg by approximately 46% and 26% at 6 and 24 h, respectively. However, the drug is currently recommended to be co-administered with a xanthine oxidase inhibitor, e.g., allopurinol or febuxostat. Combination of lesinurad 200 mg with a xanthine oxidase inhibitor results in an additional 25% and 19% decrease in serum uric acid levels at 6 and 24 h post dose, respectively [45]. To date, lesinurad has predominantly been observed to exchange lactate for uric acid, and under this treatment, increased serum lactate concentrations should be taken into account.

Probenecid, benzbromarone or sulfinpyrazone are other less selective agents inhibiting URAT1 activity, which could potentially be considered for the treatment of gout. However, not all of these agents are advised as first-line drugs. The most frequently considered for this application is probenecid, which targets URAT1 and GLUT9 and decreases reabsorption functions by 80–95%, as well as inhibiting OAT1 and OAT3 in the proximal tubule cell, leading to reduced uric acid uptake from blood. The latter activity, i.e., inhibition of OAT1 and OAT3, may contribute to retention of uric acid, thus limiting clinical applications. Sulfinpyrazone is also known to inhibit uric acid reabsorption, mainly by inhibition of URAT1, but also interacts with the basolateral aspect of the renal epithelial cells, thus affecting reabsorption into the circulation via inhibition of OAT4 and GLUT9 [46]. Benzbromarone has been withdrawn from the European market by the manufacturer (due to potential hepatotoxicity), but is still available in some countries. The drug reduces URAT1 and GLUT9 urate transport in vitro by 80–95%, decreasing the reabsorption activity, which results in decreased systemic levels of uric acid.

Some other medications, principally not registered in the treatment of gout, were defined as reducing uric acid levels via interactions with renal transporters. Angiotensin receptor antagonists, above all, losartan interact with OAT3, MRP4, URAT1 and GLUT9 and lower uric acid levels. In vitro studies demonstrated that losartan provides about 85% inhibition of URAT1 and a 70% decrease in the activity of GLUT9, leading to an increase in the fractional excretion of uric acid [47]. The increase is rather moderate, from 3% to 30%. In some studies, repeated dosing with losartan resulted in a gradual waning of the uricosuric effect, but a moderate reduction in serum uric acid was maintained after 3–4 weeks of treatment [48]. However, when elevated levels of uric acid or gout are diagnosed during treatment with other sartans (AT1, angiotensin type 1 receptor blockers), there is no recommendation to switch medication to losartan. Fenofibrate, a hypolipemic agent with a complex mode of action (mainly activation of lipoprotein lipase), was also demonstrated to increase urinary excretion of uric acid through inhibition of URAT1 by fenofibric acid, its major metabolite [49]. Lowering of serum uric acid levels was clinically significant, ranging across studies from 20% to 46%. To date, the uricosuric activity was not observed by another fibrate, bezafibrate, despite similar activity in decreasing serum lipids, and therefore does not seem to be related to the lipid lowering effects of fibrates [50]. Therefore, losartan can be considered the preferred option in the treatment of patients with elevated uric acid levels/gout and arterial hypertension, while fenofibrate is preferred in patients with dyslipidemia and hyperuricemia. Atorvastatin, another hypolipidemic agent, but not the other statins, was evidenced to reduce serum uric acid levels (by 12.5% at a dose of 40 mg/day) via uricosuric activity. However, its uricosuric mode of action was not defined [51].

Targeting renal transporters can also be applied to increase outcomes of antibacterial therapy. Probenecid (OATs inhibitor) is recommended in co-administration with antimicrobial agents (penicillins and cephalosporins), which are substrates of OATs (mainly OAT1 and OAT3), in order to increase their systemic concentrations (and also to prolong dosing interval). Competition of probenecid with cephalosporines (cefuroxime, cephalexin, cefazolin, cefuroxime, cefaclor, cefotaxime, ceftazidime) and penicillins (ampicillin, amoxicillin, flucloxacillin) for OATs reduces the rate of renal tubular secretion and drug excretion. This type of interaction aims to achieve an increase in the therapeutic efficacy of antimicrobials, as a 2-fold to 4-fold drug concentration elevation has been demonstrated for various penicillins and cephalosporins. The main indication for the combined use is treatment of uncomplicated gonorrhea, syphilis and cellulitis [52,53].

Several drugs, e.g., loop diuretics and thiazide diuretics are substrates of drug transporters, which determine their concentrations at target sites and thus clinical efficacy. To gain access to the peritubular region, loop diuretics (furosemide, bumetanide, torsemide) are secreted across the proximal tubule; this action provides mainly OAT1 and OAT3 on the basolateral membrane, subsequently reaching urine filtrate and then targeting molecules located in the thick ascending limb cells, i.e., Na-K-2Cl (NKCC) transporter. OAT1 and OAT3 have avid affinity for loop diuretics and free diuretics from albumin (OAT2 expressed on basolateral membrane of kidney epithelial cells has lower affinity for loop diuretics) [54]. The tubular concentration of a loop diuretic determines its natriuretic effect [55]. In vitro findings revealed that furosemide is also a substrate of MRP4, and might transport the drug across the apical membrane to tubular fluid [56]. Likewise, thiazides (hydrochlorothiazide, chlorothiazide, chlortalidone) are also substrates of OATs [57,58], which facilitate transport of the drugs to tubular filtrate, and then inhibit the apical sodium/chloride transporter in epithelial cells of the distal convoluted tubules. Preliminary data suggest that hydrochlorothiazide is also a substrate of MRP4, and shifts the drug from the tubule epithelial cell to the nephron lumen. Some experimental findings also suggest that the drug can reach tubule filtrate via secondary pathway, i.e., through hOCT2/hMATE2-K co-operation [59].

## 3. Kidney Carriers and Transporters and Nephrotoxicity

Activity of drug transporters in the kidney tubule epithelial cells can also play an important role in nephrotoxicity, mediating drug accumulation within the cells. Cisplatin is an illustrative example of nephrotoxicity. Only cisplatin induces nephrotoxicity among platinum anticancer drugs. The platinum compounds, being cationic agents, are predominantly handled by OCTs and MATEs in the kidney, and the interplay between transporters determines drug concentration in the tubular epithelia. The renal accumulation of cisplatin is much greater than that of the other platinum compounds. Cisplatin and oxaliplatin are carried intracellularly, mainly by OCT2 (oxaliplatin also weakly by OCT3) [60,61]. Afterwards, only oxaliplatin is shifted from the cells by MATE1 and MATE2-K, which reduce intracellular exposure to the drug, whereas cisplatin accumulates inside the tubular epithelia as it is not the efflux transporters substrate. Thus, a deficit in cisplatin efflux leads to intracellular drug accumulation and its nephrotoxic effects [60,62]. Carboplatin and nedaplatin are not substrates for OCT1, OCT3, MATE1 or MATE2K, and thus do not accumulate in kidney tubule epithelial cells [60]. Therefore, substrate specificity regulates the nephrotoxic characteristics of platinum agents.

OATs are also engaged in the nephrotoxic activity of cidofovir, which is reflected in its clinical guidelines. The OAT1 transports cidofovir intracellularly, which promotes its nephrotoxicity. To minimize drug-induced nephrotoxicity, the summary of product characteristics (SmPC) recommends oral probenecid (and intravenous saline prehydration) administration. Probenecid, as stated above, inhibits OAT1 and OAT3 in the proximal tubule cells, leading to reduced drug uptake. The SmPC directly specifies that concomitant use of probenecid is essential for reducing the pronounced nephrotoxicity of cidofovir to an extent that results in an acceptable benefit/risk balance of cidofovir therapy. Cidofovir administration is contraindicated in patients unable to receive probenecid [63]. The information in SmPC clearly highlights the importance of OATs contribution to the nephrotoxicity of this drug.

Beta-lactams, e.g., penicillins and cephalosporins are also substrates of OATs, and demonstrate mild nephrotoxic activity. Current clinical guidelines indicate probenecid, an agent that inhibits renal excretion of beta-lactam drugs, leading to their increased plasma concentrations. This strategy is recommended in clinical guidelines. However, in the case of beta-lactams more cytotoxic to the proximal renal tubule, like cephaloridine, co-administration of probenecid has been advised to prevent cytotoxicity [64]. Likewise, imipenem (beta lactam carbapenem antibiotic) itself could not be marketed, owing to its instability and nephrotoxicity, until cilastatin, an inhibitor of renal dehydropeptidase-I, was developed. Cilastatin seems to not only be an inhibitor of drug degradation by dehydropeptidase, but can also inhibit renal OATs. It has been demonstrated that cilastatin reduces intracellular accumulation of imipenem and alleviates drug nephrotoxicity [65].

Methotrexate is also a substrate of renal OATs, i.e., OAT1 and OAT3, and the carriers mediate intracellular drug accumulation, which potentially determine its nephrotoxic effects (as well as clearance in co-operation with apical efflux transporters, MRP4 and BCRP) [66]. Some experimental studies suggest that inhibition of OAT function (e.g., by rhein, glucuronides) reduces nephrotoxic potential of methotrexate [67,68].

## 4. Kidney Transporters and Drug Elimination

Interplay between uptake carriers and efflux transporters determines vectorial transport of drugs across kidney epithelial cells, and thus mediates drug elimination.

In the kidney, OCT2 operating on the basolateral membrane works in concert with the luminal-facing MATE1 and MATE2-K, and thus mediates active renal secretion of organic cations. OCT2 and MATE1 or MATE2-K double-transfected Madin–Darby canine kidney cells revealed clear directional transport of procainamide and quinidine [69]. There is also evidence that these transporters are engaged in the elimination of metformin [70] and atenolol [71]. Looking at the affinity of various drugs to human MATE1, MATE2-K and OCT2, it can be suggested that cimetidine, chlorpheniramine, diphenhydramine, disopyamide, famotidine, pramipexole, procainamide, quinidine, quinine and verapamil can also be eliminated in the kidney via the cation transporter system co-operation [70].

Similar to the cation transporter system, findings demonstrate coordinated transport of organic anion drugs in the human kidney. Co-expression of OAT1, OAT3 on the basolateral membrane and MRP2, MRP4 on the apical membrane of proximal epithelial cells [72] suggests their involvement in vectorial drug transport. Estradiol-17beta-d-glucuronide and methotrexate are substrates of both OATs and MRPs [73,74], and the transporters could be involved in their elimination. In general, drug conjugates are substrates for both OATs and MRPs, and the co-operation between the uptake and efflux transporters may contribute to conjugated compounds renal elimination [75]. It also seems that the OAT1 (OAT3)/MRP4 system can play an important role in antiviral (adefovir, zidovudine) and antibacterial (benzylpenicillin) agents’ renal excretion [76,77,78]. Another anion transport system can be suggested for digoxin handling in the proximal tubule epithelial cells. Experimental data, along with the PBPK kidney model for digoxin, suggest the involvement of apical OATP4C1 and basolateral P-gp in tubular secretion [79].

Immunohistochemistry analysis revealed localization of BCRP in the proximal tubule brush border membrane in the human kidney. However, proteomic studies revealed its very low protein abundance (immunohistochemistry is a more sensitive, but less specific method in comparison to targeted proteomics) [8,9]. The function of BCRP in the kidney was positively verified by fumitremorgin C and nelfinavir (BCRP inhibitors)-mediated inhibition of Hoechst 33,342 dye efflux from primary human proximal tubule cells [80]. These findings may suggest that BCRP may contribute to organic cation excretion in the kidney.

## 5. Kidney Carriers and Transporters and Drug–Drug Interactions

Clinical studies also provide information about DDIs at the site of renal carriers and transporters. Some DDIs are presented above in Section 2 “Kidney Transporters in Pharmacotherapy”, and are presented in Table 2 (for drugs with information about renal clearance). Inhibition of basolateral uptake carriers contributes to reduction in renal secretion clearance of drugs, which may result in increased concentrations in blood, whereas decrease in the apical transporter functions can produce decrease in elimination rate, but also may lead to drug accumulation in the proximal tubule cells leading to drug-induced nephrotoxicity.

Metformin is an example of cationic drug with DDIs in the kidney. The drug, nowadays, is the first line oral treatment for type 2 diabetes, and is a substrate of OCT2/MATE1/-2K. The interaction between cimetidine (perpetrator) and metformin (victim) was first ascribed to inhibition of OCT2, but more recent studies suggest a predominant role of MATE1 in the explanation of clinical study findings on the DDI. It was demonstrated (in vitro) that cimetidine had much greater inhibition potency to MATE1/2-K (Ki 1.1–6.9 mmol/L) than for OCT2 (Ki 95–146 μmol/L) [81]. From a mechanistic point of view, it seems that OCT2-mediated uptake of cimetidine into kidney epithelial cells could also produce an impact on the drug inhibitory activity at MATE1/2-K sites [82]. Similarly to cimetidine, DDI between metformin (victim) and pyrimethamine (perpetrator) depends on the inhibition of MATE1/2-K transporter, as pyrimethamine potency against MATE1/2-K is about 100-fold higher than towards OCT2. This interaction leads to a significant 1.4-fold increase in metformin area under the concentration-time curve (AUC) and 35% decrease in clearance [83]. The relatively small magnitude of the DDI can be attributed to high protein binding of pyrimethamine, which results in small unbound concentration and low diffusion ability to tubular epithelia cells. However, OCT2 seems to play a predominant role in metformin DDI with dolutegravir. Dolutegravir is a more potent inhibitor of OCT2 (IC_50_ 1.9 mmol/L) than MATE1/2-K (IC_50_ 6.3–25 mmol/L). Co-administration of dolutegravir produced a 2.5-fold increase in metformin AUC (a magnitude that exceeded changes induced by the above mentioned cimetidine and pyrimethamine) [84].

DDIs were also revealed at OATs/MRPs/P-gp sites. The best characterized and explored in clinical medicine are interactions with probenecid. Probenecid inhibits OAT1 and OAT3 with similar potency, with similar Ki values of 4–12 mmol/L [6]. Inhibitory activity against apical MRP2, MRP4 and OAT4 is less potent, with Ki of 44.6, 2300, and 54.9 mmol/L, respectively [85]. Some DDIs are of therapeutic value, and are described above. Other DDIs involving probenecid include probenecid–fexofenadine or probenecid–furosemide interactions. Probenecid (perpetrator) markedly reduces furosemide (victim) and fexofenadine (victim) renal clearance and urinary excretion (and also increases systemic exposure and half-life). For furosemide, a fold increase in AUC from 2.7 to 3.1 was reported to reduce renal clearance by 66% to 80% [86,87]. However, the interaction does not entirely explain the diuretic response to furosemide, which in clinical settings has even been reported to increase, i.e., exhibiting decreased response in the first 60–90 min post dose followed by a more intense response afterwards, leading to a greater overall effect [88]. Probenecid (perpetrator), mainly via inhibition of OAT3, increased fexofenadine (victim) AUC 1.5-fold and decreased its renal clearance by approximately 70–73% [85,89]. Interactions between methotrexate and proton pump inhibitors (PPIs) are of clinical importance, since the drugs are commonly co-medicated. At clinical plasma concentrations of methotrexate, approximately 25% of renal clearance is excreted by glomerular filtration, and the remaining 75% is excreted by active tubular secretion. Clinical observations suggest that in patients administered methotrexate and PPIs, a 27% decrease in the clearance of methotrexate is noted [90]. These clinical observations can be explained by in vitro studies, which demonstrated that PPIs (perpetrators) inhibit methotrexate (victim) transport via inhibition of OAT3 function. The study also characterized human OAT3 as a high-affinity-type transporter of methotrexate, with a K_m_ value of 21.17 µM [91]. Clinical observations also evidence that combined administration of methotrexate and non-steroidal anti-inflammatory drugs (NSAIDs) can result in elevated plasma concentrations of methotrexate and severe adverse effects, such as liver injury, renal failure, gastrointestinal disorders, and myelosuppression [92,93]. Experimental findings suggest that NSAIDs produce inhibition of OAT1 and OAT3 that can contribute to the observed DDIs. The inhibitory effects of NSAID glucuronides (diclofenac, R- and S-ibuprofen, R- and S-flurbiprofen, and R- and S-naproxen) seem to be more potent (5- to 15-fold) than the parent drugs, especially on OAT1- and OAT3-mediated methotrexate transport. The IC_50_ values of NSAIDs were from 174 to 960 µM for OAT1, and 3.17 to 127 µM for OAT3 [67].

P-gp can also be a site of DDIs of clinical importance. P-gp interaction made it possible to explain the observed in clinical medicine increase in digoxin systemic concentrations when co-administered with quinidine. Digoxin is a well-established P-gp substrate, and is characterized by a narrow therapeutic index. Therefore, subtle changes in the drug concentrations may precipitate its toxic effects. Co-administration of digoxin (victim) with quinidine (P-gp substrate and inhibitor, perpetrator) produced a 33–56% decrease in renal clearance of the victim drug due to impaired P-gp mediated efflux function [85]. A similar digoxin DDI mechanism was reported for clarithromycin (perpetrator). The renal clearance of digoxin decreased by 20% with parallel significant reduction in the nonglomerular renal clearance, which could be ascribed to inhibition of P-gp-dependent renal tubular transport of digoxin. Assuming that renal clearance accounts for approximately 80% of total digoxin clearance, 20% decrease in renal clearance was expected to result in only a 1.14-fold increase in digoxin AUC, which was observed in the pharmacokinetic study [94]. A similar decrease in the renal clearance of digoxin was observed during coadministration of itraconazole [95], which is a potent inhibitor of P-gp [96]. Verapamil, a short-term inhibitor of mainly P-gp, has been used to increase the therapeutic effectiveness of cytotoxic anticancer drugs in cancer chemotherapy. Likewise, this activity in the kidney can lead to DDIs. The interaction at P-gp site in the kidney can explain clinically important DDI between verapamil and digoxin. Verapamil (perpetrator) resulted in a significant 1.5-fold reduction in total body clearance of digoxin (victim). The decline in digoxin total body clearance was partially caused by a 21% decrease in renal clearance and partially by a 60% impairment of extrarenal clearance [97]. The same interaction mechanism, i.e., P-gp function inhibition, may precipitate interaction between verapamil and fexofenadine (P-gp substrate). Verapamil treatment was evidenced to significantly increase the fexofenadine peak plasma concentration by 2.9-fold and AUC by 2.5-fold, but the interaction can also depend on P-gp inhibition in the intestine and increased bioavailability [89,98].

The function of breast cancer resistance protein (BCRP/ABCG2) in human kidney remains a bit controversial, as stated above. The low level of expression and paucity of clinical verification rather suggest a limited role of renal BCRP in DDIs. The latter statement is based on the recently published review, which concluded after analyzing the available information that there is a lack of clinical evidence for the involvement of BCRP in the renal clearance of substrate drugs [98]. It is important to highlight that the lack of clinical evidence does not prove that there is not a DDI, particularly considering that changes in local renal cell concentrations of drugs may not be reflected in systemic concentrations.

**Table 2:** DDIs involving renal drug transporters in human studies.

Transporter/s Involved	Victim Drug	Perpetrator Drug	AUC Fold Increase	CL_R_ Decrease (%)	Ref.
P-gp	Digoxin	Quinidine	-/-	56/33	[99,100]
	Digixin	Clarithromycin	1.1	20	[94]
	Digoxin	Verapamil	-	21	[97]
	Digoxin	Itraconazole	1.5	20	[95]
OCT2, MATE1, MATE2-K	Metformin	Cimetidine	1.5/1.5	28/45	[101,102]
	Metformin	Pyrimethamine	1.4	35	[83]
	Gemifloxacin	Cimetidine	1.1	28	[103]
	Fexofenadine	Cimetidine	1.3	34	[89]
	Zidovudine	Trimetoprim	-	48	[104]
	Zidovudine	Trimetophrim/sulphamethoxazole	-	59	[104]
OAT1, OAT3	Furosemide	Probenecid	2.7/3.1	66/80	[86,87]
	Fexofenadine	Probenecid	1.5/1.5	73/70	[89,105]
	Acyclovir	Benzylpenicillin	1.3	56	[106]
	Mycophenolate mofetil	Acyclovir	1.3	11	[107]
	Cidofovir	Probenecid	1.8	52	[52]

AUC—area under the concentration-time curve; CL_R_—renal clearance.

## 6. Conclusions

Approximately 30% of the top 200 prescribed drugs in the U.S. in 2010 are eliminated by the kidney, with a fraction excreted unchanged in urine of more than 0.25. Among the mainly renally eliminated drugs, 92% undergo net secretion in which transport system is engaged [5]. Therefore, information about drug transporters in the kidney is important in clinical drug applications. The kidney tubule epithelia cells express membrane carriers and transporters, which play an important role in in drug handling and pharmacotherapy as it is evidenced in the literature. The renal tubule transport systems are involved in drugs (and metabolites) elimination, drug-induced nephrotoxicity, drug–drug interactions as well as constitute direct drug targets. Despite significant progresses in the understanding of renal drug transporters, the information about the regulation mechanisms affecting the function is still limited. The proteomic data on renal drug transporters are scarce, and no proteomic information on renal drug transporters in human kidney pathology is available. Accumulating information suggests that intact nephron hypothesis (assuming that the total renal excretion clearance of a drug declines proportionally with GFR) is not entirely operating as tubular secretion and re-absorption rather do not decrease proportionally to GFR. The recent analysis estimated 50% decline in OAT1/3 activity beyond the CKD-related changes in GFR [19,108] The OCT2 activity seems rather to decline in parallel with the severity of CKD [109]. These findings highlight the inadequacy of intact nephron hypothesis assumptions. Having in view the recent reports, it can be suggested that for mild CKD the use of intact nephron hypothesis is adequate; in moderate CKD, reduction in non-renal clearance should be considered, and in addition to these two adjustments a further reduction in intrinsic clearance mediated by drug transporters (especially OATs) is required for predicting drug pharmacokinetics in severe CKD [108,110]. The functional role of transporters in the kidney is also affected by drug protein binding (albumin or α1-acid glycoprotein), the changes of which in CKD are well-established [19], or interactions of transporters with uremic toxins/solutes (e.g., indoxyl sulfate, p-cresyl sulfate, kynurenine) [111,112]. The adaptive transporter changes in other organs during CKD can also modify drug pharmacokinetics and DDIs [113].

A list of defined substrates (e.g., Table 1) as well as inhibitors of renal membrane carriers and transporters suggests their implications in drug pharmacokinetics and DDIs. However, clinical studies not always confirm possible engagement of renal transporters, e.g., a recently published analysis indicates limited clinical DDIs risk upon P-gp or BCRP inhibition in the kidney [98]. The possible differences between actual concentrations of substrates/inhibitors at the transporter protein site (difficult to measure) and plasma concentrations may affect reliable analysis of study findings. The substrate-dependent and time-dependent inhibitions can be other confounding factors [114,115].

It is known that nuclear receptor-mediated transcriptional and epigenetic regulation mechanisms determine expression of renal drug transporters. However, the regulation mechanisms of drug transporters in other organs (liver, intestine) are better defined than in the kidney [26]. There are scarce data on transcriptional and post-transcriptional mechanisms involved in renal transporter regulation, with few information about regulatory role of uremic solutes/toxins. Furthermore, most of the findings are from experimental studies, and information on regulation of drug transporters in the human kidney still needs to be defined.

In summary, based on the evidence presented, it can be stated that kidney tubule epithelia cells express membrane carriers and transporters, which play an important role in pharmacotherapy. The renal tubule transport systems are involved in pharmacokinetics, drug-induced nephrotoxicity, DDIs as well as constitute direct drug targets. A list of defined drug substrates (see Table 1) as well as inhibitors of the transporters suggests even more important role than presented of the kidney transporter system in pharmacotherapy, but a direct verification of its involvement should be provided in future studies.

## Figures and Tables

**Table 1:** Selected drug substrates of the principal renal transporters [13,14,15,16].

Transporter	
Gene/Protein	Drugs
ABC transporters	
*ABCB1*/MDR1 P-glycoprotein	actinomycin D, amitriptyline, amprenavir, atorvastatin, carbamazepine, celiprolol, clopidogrel, citalopram, colchicine, cyclosporin A, daunorubicin, dexamethasone, digoxin, diltiazem, doxycycline, doxorubicin, erythromycin, etoposide, fexofenadine, imatinib, indinavir, irinotecan, itraconazole, ketoconazole, lamotrigine, lansoprazole, levetiracetam, levofloxacin, loperamide, losartan, lovastatin, melphalan, methylprednisolone, mitomycin C, mitoxantrone, morphine, nelfinavir, omeprazole, ondansetron, paclitaxel, pantoprazole, phenobarbital, phenytoin, propanolol, quinidine, rifampicin, ritonavir, saquinavir, simvastatin, sirolimus, sparfloxacin, tacrolimus, talinolol, 99mTc-MIBI, teniposide, tetracycline, topotecan, vecuronium, verapamil, vinblastine, vincristine
*ABCC2*/MRP2	adefovir, ampicillin, azithromycin, ceftriaxone, cidofovir, cisplatin, cyclophosphamide-GS, doxorubicin, doxorubicin-GS, epirubicin, estradiol 17βD-G, etoposide-G, etoposide, hydroxynonenal-GS, hyodeoxycholate-G, indinavir, irinotecan, lopinavir, melphalan-GS, methotrexate, mitoxantrone, nelfinavir, olmesartan, ritonavir, saquinavir, SN-38-G (irinotecan metabolite), valsartan, vinblastine, vincristine
*ABCC4*/MRP4	6-mercaptopurine, 6-thioguanine, acyclovir, adefovir, cefazolin, ceftizoxime, furosemide, hydrochlorothiazide, leucovorin, methotrexate, olmesartan, PAH, para-methoxy-N-ethylamphetamine, ritonavir, tenofovir, topotecan
*ABCG2*/BCRP	6-mercaptopurine, 6-thioguanine, acyclovir, adefovir, cefazolin, ceftizoxime, furosemide, hydrochlorothiazide, leucovorin, methotrexate, olmesartan, PAH, para-methoxy-N-ethylamphetamine, ritonavir, tenofovir, topotecan
**SLC carriers**	
*SLC22A2*/OCT2	amantadine, amiloride, cimetidine, cisplatin, D-tubocurarine, famotidine, ifosfamide, lamivudine, memantine, metformin, oxaliplatin, pancuronium, pindolol, propranolol, varenicline, zalcitabine
*SLC22A4*/OCTN1	doxorubicin, entecavir, ergothioneine, gabapentin, imatinib, metformin, mitoxantrone, oxaliplatin, pregabalin, quinidine, tiotropium, ipratropium, verapamil
*SLC22A5*/OCTN2	cephaloridine, emetine, entecavir, etoposide, imatinib, ipratropium, spironolactone, tiotropium, verapamil
*SLC22A6*/OAT1	adefovir, cephaloridin, ciprofloxacin, methotrexate, pravastatin, zidovudine
*SLC22A7*/OAT2	5-fluorouracil, acyclovir, bumetadine, diclofenac, entecavir, ganciclovir, irinotecan, PAH, penciclovir, tetracycline, zidovudine
*SLC22A8*/OAT3	adefovir, cefaclor, ceftizoxime, cephaloridine, ciprofloxacin, conjugated sex steroids, methotrexate, NSAIDs, pravastatin, zidovudine
*SLC47A1*/MATE1	acyclovir, cephalexin, cephradine, cimetidine, fexofenadine, ganciclovir, metformin, oxaliplatin, topotecan
SLC47A2/MATE2-K	acyclovir, cimetidine, ganciclovir, lamivudine, metformin, oxaliplatin, quinidine, topotecan
*SLCO4A1*/OATP4A1	digoxin, ouabain, methotrexate

Sulfate conjugates (-S), glutathione conjugates (-GS), glucuronide conjugates (-G). 99mTc-MIBI—[99mTc]methoxyisobutylisonitrile; NSAID—nonsteroidal anti-inflammatory drug; PAH—para-aminohippurate.

## Data Availability

No new data were created or analyzed in this study. Data sharing is not applicable to this article.
